# Management of Upper Gastrointestinal Bleeding by an Internist

**DOI:** 10.7759/cureus.2878

**Published:** 2018-06-25

**Authors:** Saad Saleem, Abell L Thomas

**Affiliations:** 1 Internal Medicine, Mercy St. Vincent Medical Center, Toledo, USA; 2 Gastroenterology, University of Louisville, Louisville, USA

**Keywords:** upper gi, gastrointestinal bleed, internist

## Abstract

Nonvariceal upper gastrointestinal bleeding (UGIB) carries high morbidity and mortality, which can be lowered by timely evaluation and management. This article presents a comprehensive literature review and current guidelines for the management of nonvariceal UGIB by an internist. Pre-endoscopic management includes optimal resuscitation, and making a decision about holding the anticoagulation and antiplatelet therapy versus continuation due to risk of thrombosis. Proton pump inhibitors (PPIs) are beneficial for both ulcer and nonulcer diseases as they reduce the risk of re-bleeding by clot stabilization. Endoscopy should only be performed after hemodynamic stability has been achieved and should not be delayed by more than 24 hours. Resumption of anticoagulation and antiplatelet therapy is based on endoscopic findings and thromboembolic risk. The patient should be discharged on PPIs and should be followed up by a primary care physician.

## Introduction and background

Upper gastrointestinal bleeding (UGIB) is defined as a clinically significant bleeding (no fixed amount of blood) from the gastrointestinal (GI) tract above the ligament of Treitz (anatomical landmark between the duodenum and jejunum) which includes the esophagus, stomach, and duodenum. The patient usually shows signs of bleeding in the GI tract either as hematemesis (vomiting of bright red blood or coffee ground emesis) or melena (black tarry stools). The majority of melena (black, tarry stool) originates proximal to the ligament of Treitz (90%), though it may as well arise from the oropharynx or nasopharynx, small bowel, or right colon [1].

Melena may be seen in varying stages of blood loss, with as little as 50 ml of blood [2]. Hematemesis (vomiting of bright red blood) is suggestive of moderate to severe bleeding, whereas coffee ground emesis (oxidation of the heme molecule of red blood cells due to gastric acid) suggests more limited bleeding. The yearly incidence of hospitalization for acute UGIB is about 100 per 100,000 people [3]. The UGIB rate of admission to the hospital is six times higher as compared to lower GI bleeding [3].

Although the diagnosis and management of nonvariceal UGIB has improved over time, it still results in 400,000 hospital admissions per year, costing more than two billion dollars per year [4]. Despite advances in therapy, hospital mortality resulting from UGIB remains high, which can be lowered by proper evaluation and management. The reported frequencies of specific causes have changed over time. Peptic ulcer disease constitutes roughly 20%-25% of cases as compared to older studies when it used to constitute half of UGIB [5-7]. The most common causes of UGIB include the following (in approximate descending order of frequency) [5-6, 8-10]: gastric and/or duodenal ulcers, esophagogastric varices, severe or erosive esophagitis, severe or erosive gastritis/duodenitis, portal hypertensive gastropathy, angiodysplasia (also known as vascular ectasia), mass lesions (polyps/cancers), Mallory-Weiss syndrome, and no lesion identified (10%-15% of patients).

The source of bleeding cannot be recognized in 10%-15% of patients with UGIB; either the lesion is hard to identify (such as a Dieulafoy's lesion), obscured by a retained blood clot at endoscopy, or the lesion has already healed by the time endoscopy was performed. The four major risk factors for bleeding peptic ulcers are [11-12]: *Helicobacter pylori* (*H. pylori*) infection, use of nonsteroidal anti-inflammatory drugs (NSAIDs), physiologic stress, and excess gastric acid.

## Review

Pre-endoscopy management

Patients are triaged based on hemodynamic status, age, comorbidities, and initial lab results. The first and foremost step in the management of UGIB is assessing the hemodynamic status and starting a resuscitative measure. In acute UGIB, hemoglobin is not a good indicator for estimating GI blood loss. The patient should receive intravenous (IV) isotonic fluids, and transfusion should be given maintaining the hemoglobin at a level ≥7 g/dl (70 g/l) or above if the patient is symptomatic [13].

Risk assessment regarding further GI bleeding and mortality will determine decisions regarding level of care, timing of endoscopy, and discharge planning. Main scores are the Blatchford score, Rockall score, and AIMS65. These scores are used to identify patients with UGIB who need to be admitted to the hospital, as the bleeding requires further medical intervention such as a blood transfusion or an endoscopic intervention. The Blatchford score as compared to the Rockall score does not take endoscopic findings into account, so it can be calculated after the patient comes to the emergency room. This score is based upon the blood urea nitrogen, hemoglobin, systolic blood pressure, pulse and presence of melena, syncope, hepatic disease, and/or cardiac failure. The score ranges from 0 to 23, and the chances of requiring an endoscopic intervention increases with a higher score. Meta-analysis has shown that a score of 0 was associated with a low likelihood of needing an endoscopic intervention [14].

The AIMS65 studies have suggested that it has a high accuracy for predicting inpatient mortality among patients with UGIB. It was derived from a database of 187 US hospitals [15]. Studies have shown that an increasing score is associated with higher mortality and a prolonged stay in the hospital. The study found that five factors were associated with increased inpatient mortality [15]: albumin less than 3.0 g/dl (30 g/l), international normalized ratio (INR) greater than 1.5, altered mental status, systolic blood pressure of 90 mmHg or less, and age older than 65 years.

Anticoagulation and antiplatelet therapy should be performed for patients with UGIB. However, the risk of thrombosis should be weighed against the risk of bleeding. Anticoagulation should be reversed in case of acute hemorrhage. If a patient taking warfarin and INR is supratherapeutic, fresh frozen plasma (FFP) or prothrombin complex concentrate should be given. There is no antidote for most of the newer anticoagulation agents, but they have a short half-life in the presence of normal kidney function. These medications should be held and bleeding will likely stop in the next 12-24 hours [16]. A small study (conducted in 1994) that included 52 patients showed successful hemostasis after endoscopic therapy in 91% of patients after correcting the INR to 1.5-2.5 compared to the control population who were not anticoagulated [17].

Proton pump inhibitors (PPIs) should be started in patients admitted with UGIB until the source of bleeding is identified. It is beneficial in both ulcer and nonulcer diseases. A high-dose intravenous infusion of PPI reduces the risk of re-bleeding ulcers [18]. It promotes hemostasis by neutralizing gastric acid, which leads to clot stabilization [19]. Gastroenterology service should be involved in all the patients with significant UGIB. There is data supporting the use of IV erythromycin before endoscopy. It improves visualization of the stomach by moving the blood and food particles from the stomach [20], thereby increasing the chances of visualizing the bleeding vessel. It reduces the need for a second-look endoscopy. 

The patient’s discharge to home directly from the emergency room (ER) without inpatient endoscopy may be considered if urea nitrogen < 18.2 mg/dl, hemoglobin greater than or equal to 13 g/dl for men and 12 mg/dl for women, systolic blood pressure greater than or equal to 110 mmHg, pulse < 100/min, absence of melena, syncope, cardiac failure, and liver disease as they have less than one percent chance of requiring intervention [13]

Upper endoscopy

Endoscopy should be performed in a nonemergent setting. Patients should be hemodynamically stabilized and should undergo an endoscopy within 24 hours of admission [13]. If the patient is hemodynamically stable on admission and does not have serious comorbidities, an endoscopy should be performed as soon as possible [13]. Patients with endoscopic findings of high-risk stigmata (Table 1) (active bleeding, visible vessel, clots) should be hospitalized for three days assuming no further episode of bleeding occurs. They can be fed with clear liquids soon after endoscopy [13]. Clear liquids provide the advantage that if the patient starts to bleed again, sedation and anesthesia can be given within two hours after the last ingestion [21]. Patients with clean-based ulcers can be discharged home if they have a residence and someone can observe them [13].

**Table 1:** Endoscopic predictors of recurrent peptic ulcer hemorrhage [22]

Endoscopic stigmata of recent hemorrhage	Prevalence (%)	Risk of re-bleeding on medical management (%)
Active arterial bleeding (Forrest Ia)	10	90
Oozing without visible vessel (Forrest Ib)	10	10-20
Non-bleeding visible vessel (Forrest IIa)	25	50
Adherent clot (Forrest IIb)	10	25-30
Flat spot (Forrest IIc)	10	7-10
Clean ulcer base (Forrest (III)	35	3-5

The risk of bleeding due to endoscopic procedures is classified as low (diagnostic procedures) or high (therapeutic procedures) (Table 2). For high-risk procedures and low thromboembolic risk, the recommended duration for discontinuing the agent before the procedure is as follows: five days (clopidogrel), three to five days (ticagrelor), seven days (prasugrel), or 10-14 days (ticlopidine) [23-25]. Stopping P2Y12 platelet receptor blockers should also be considered in patients at high risk for thromboembolic complications who are undergoing high-risk procedures, if the risk of bleeding is thought to outweigh the risk of thromboembolism. Aspirin need not be discontinued. A cardiologist or a neurologist should be consulted based on the indication of the antiplatelet therapy.  

**Table 2:** The management of antithrombotic agents for patients undergoing gastrointestinal endoscopy *Abbreviations*: EGD, esophagogastroduodenoscopy; ERCP, endoscopic retrograde cholangiopancreatography; PEG, percutaneous endoscopic gastrostomy; EUS, endoscopic ultrasound; FNA, fine-needle aspiration; EMR, endoscopic mucosal resection; PEJ, percutaneous endoscopic jejunostomy [26]

Higher-risk procedures
Polypectomy
Biliary or pancreatic sphincterotomy
Treatment of varices
PEG placement
Therapeutic balloon-assisted enteroscopy
EUS with FNA
Endoscopic hemostasis
Tumor ablation
Cystogastrostomy
Ampullary resection
EMR
Endoscopic submucosal dissection
Pneumatic or bougie dilation
PEJ
Low-risk procedures
Diagnostic (EGD, colonoscopy, flexible sigmoidoscopy) including mucosal biopsy
ERCP with stent (biliary or pancreatic) placement or papillary balloon dilation without sphincterotomy
Push enteroscopy and diagnostic balloon-assisted enteroscopy
Capsule endoscopy
Enteral stent deployment (controversial)
EUS without FNA
Argon plasma coagulation
Barrett's ablation

The American Society of Gastrointestinal Endoscopy (ASGE) guidelines recommend avoiding elective procedures on cardiac stents patients until they has received antithrombotic therapy for the minimum recommended duration based upon the current guidelines [26]. Once beyond that period, depending on the type of stent used, P2Y12 platelet receptor blockers are discontinued 5-14 days prior to the procedure, while continuing aspirin. The guidelines recommend that patients who are within six weeks of bare-metal stent placement or within six months of drug-eluting stent placement have surgical procedures deferred, if possible, until this period [26]. After the procedure, the P2Y12 platelet receptor blocker should be restarted, with or without a loading dose, as soon as it is considered safe, depending on the patient's indication for taking it and any intervention performed during the endoscopy that may increase the risk for bleeding (such as removal of a large polyp) [27].

Post-endoscopy 

 Data are limited about resumption of anticoagulation after achieving endoscopic hemostasis, but studies have shown that resuming anticoagulation has better outcomes than not resuming. Unfractioned heparin is preferred for bridge therapy due to a shorter half-life of 1.5 hours [26]. After endoscopy, the patient should be followed up for the results of *H. pylori*. Patients with *H. pylori* associated bleeding ulcers should receive *H. pylori* therapy. Once the eradication is documented, they do not require long-term antisecretory therapy [13]. A patient who develops NSAID-associated ulcers should be carefully assessed. If needed to be resumed, the lowest dose cyclooxygenase-2 (COX-2) selective NSAID with daily PPI is recommended [13]. If the patient develops low-dose aspirin-induced ulcers, aspirin has to be stopped if given for primary prevention of cardiovascular disease. It can be continued if given for secondary prevention of cardiovascular diseases with long-term use of PPI. Aspirin can be resumed between three and seven days after UGIB [13]. The PPI should be continued in the long term if the gastric ulcer is idiopathic. Aspirin and other NSAIDS given alone in standard doses do not increase the risk of bleeding after an upper endoscopy with biopsy or biliary sphincterotomy [28-31].The data are conflicting about whether aspirin or/and NSAIDS increase the risk of bleeding postpolypectomy.

The 2010 International Consensus Recommendations do not recommend routine use of a second-look endoscopy for nonvariceal UGIB [32]. The guidelines suggest that patients at a particularly high risk for recurrent bleeding may benefit from a second-look endoscopy; these patients include those whose first endoscopy was limited or if the first endoscopic therapy was suboptimal.

 A physician should monitor the patients for the following which may suggest re-bleeding [33]: hematemesis more than six hours after the initial endoscopy, melena after normalization of stool color, hematochezia after normalization of stool color, development of tachycardia (heart rate ≥110 beats per minute) or hypotension (systolic blood pressure ≤90 mmHg) after one hour of hemodynamic stability in the absence of other possible alternatives, hemoglobin drop of 2 g/dl or more after two consecutive stable hemoglobin values with at least three hours difference, and tachycardia or hypotension that does not resolve within eight hours. Patients with signs of recurrent bleeding following first endoscopic therapy are typically treated with a second endoscopic therapy.

Physicians should be diligent in avoiding the complications associated with endoscopy. Complications are more common with emergent endoscopy [34]. It includes aspiration pneumonitis, hypoventilation due to oversedation, or hypotension due to inadequate volume replacement in addition to sedation with opiates. Postoperative complications include perforation of the esophagus leading to mediastinitis; epinephrine injections can cause tachycardia and arrhythmias [34].

Long-term use of PPI has been associated with several side effects. Its use has been associated with increased risk of Clostridium difficile infection in the absence of antibiotic use [35,36]. Its use has been associated with microscopic colitis, including lymphocytes and collagenous colitis [37]. PPI can increase the risk of fractures. Induced hypochlorhydria can augment osteoclastic activity, thereby decreasing bone density [38]. PPI can cause acute interstitial nephritis [39]. Patients should follow up with a primary care physician after discharge to decide about PPI.

## Conclusions

Upper GI bleeding is a medical emergence with high mortality which can be lowered by proper assessment and management. A validated scoring system can help the internist decide about the level of care, timing of endoscopy, and discharge planning. The risk of thrombosis ought to be weighed against the risk of bleeding before holding the anticoagulation and antiplatelet therapy in UGIB. Endoscopy should be performed after hemodynamically stabilizing the patient. It should be performed within 24 hours of admission. Data are restricted about resumption of anticoagulation after accomplishing endoscopic hemostasis. It should be individually based on anticoagulation indication and on endoscopic findings. In spite the fact that PPI utilization brings down the risk of re-bleeding, long-term use of PPI should be justified considering the side effects related with it.
